# Current Landscape of Mental Health Conversational Agents From a Trauma-Informed Care Lens: Scoping Review

**DOI:** 10.2196/77876

**Published:** 2026-04-30

**Authors:** Faye Kollig, Kira Voelker, Emily Ryan, Rachel Pfafman, Fayika Farhat Nova

**Affiliations:** 1 Department of Information Science, University of Colorado Boulder, Boulder, CO, United States; 2 Parkview Research Center, Health Services and Informatics Research, Parkview Health, 10622 Parkview Plaza Dr, Fort Wayne, IN, 46845, United States, (260) 266-7701

**Keywords:** conversational agents, trauma-informed care, mental health, chatbots, artificial intelligence, human-AI interaction

## Abstract

**Background:**

Conversational agents (CAs) are increasingly used in mental health care to enhance access and engagement. However, their safe, ethical, and user-sensitive design remains a challenge. Despite growing attention to trauma-informed approaches in human-computer interaction, there is limited work on how the trauma-informed care (TIC) framework could be applied in the design of mental health CAs and no comprehensive synthesis to date.

**Objective:**

Guided by the Substance Abuse and Mental Health Services Administration’s TIC framework, this scoping review explored how TIC principles (safety; trustworthiness and transparency; collaboration and mutuality; empowerment, voice, and choice; peer support; and cultural, historical, and gender issues) are currently represented in the design and evaluation of mental health conversational agents (MHCAs) and identified gaps and opportunities to promote more trauma-informed design practices.

**Methods:**

Online databases, as well as a secondary survey of citation lists from an initial search, were used to identify English-language journal articles and conference proceedings from 2000 to 2024 that empirically evaluated an independent, web- or app-based, unassisted CA used for mental health and included concepts from TIC.

**Results:**

Our analysis included 38 publications (n=28, 73.7%, published in 2020 or later) covering 28 distinct MHCAs. Most studies used experimental methods (n=23, 60.6%) or user studies (n=11, 28.9%), with samples skewed toward female (men: mean 34.92%, SD 18.64%), young in age (mean 32.52, SD 14.6 y), and predominantly nonclinical (n=29, 76.3%). MHCAs were largely rule-based prototypes. No studies explicitly referenced the TIC framework as a guiding lens for MHCA design or evaluation. A total of 26 studies referenced terminology from TIC core principles but rarely defined them, while all 38 included language that could be linked to one or more principles. Overall, TIC-related concepts appeared most often within intervention design descriptions, qualitative assessments, or as items embedded in questionnaires evaluating broader constructs. Trustworthiness and transparency, safety, empowerment, voice and choice, and collaboration and mutuality were comparatively well addressed, while peer support and cultural, historical, and gender issues were largely absent. Design recommendations, where present, were relatively broad and emphasized secure, customizable, reliable, human-like, and context-sensitive MHCAs that offered multimodal interaction, goal setting and tracking, and transparency.

**Conclusions:**

Studies did not self-identify as using Substance Abuse and Mental Health Services Administration’s framework for TIC, making it more difficult to identify its elements. The fragmented terms, disciplines, and metrics used make it difficult to draw more systematic conclusions about the current research landscape related to TIC, but our analysis indicates TIC to be a descriptive and potentially unifying framework and provides a starting point for the explicit trauma-informed MHCA research and design.

## Introduction

Conversational agents (CAs), or chatbots, are digital systems designed to engage users in interactive exchanges through text, voice, or visual interfaces [1]. CAs are designed to simulate human-to-human interactions, reimagined as human-machine dialogues [2]. In recent years, CAs have been increasingly adopted in mental health settings for their potential to enhance access and engagement, sustaining the use of digital interventions [1,3]. When used in the context of mental health, CAs aim to deliver support and even psychological interventions, blurring the line between digital convenience and therapeutic care [4-6]. There are 2 approaches that are used to design chatbots: rule-based and machine learning (ML)–based [7]. As artificial intelligence (AI) continues to evolve at an unprecedented pace [8], mental health CAs (MHCAs) have become both a frontier of innovation and a matter of ethical concern.

While early reviews highlight promising outcomes from CA-based mental health interventions, the evidence remains mixed and context-dependent [1,9,10]. Studies have shown that MHCAs are valuable for conducting private conversations [11], aiding in learning [12], improving users’ well-being [13], preparing them for interactions with health care providers [14], and boosting their self-efficacy [1,11]. Reflecting this growing interest, the MHCA ecosystem has expanded into a multibillion-dollar market, with widely adopted tools such as Wysa, Woebot, and Youper [3,15,16]. These systems commonly draw on evidence-based therapeutic approaches, including cognitive behavioral therapy (CBT), mindfulness, positive psychology, and psychoeducation, to deliver scalable mental health support to users [3].

However, recent instances of critical failures of CAs to properly and ethically support end users have ignited public scrutiny [17,18], raising questions on how to design, evaluate, and implement these tools in the mental health domain. On some occasions, users have reported that their interactions with CAs were distressing [13] or approximated sexual harassment [19] or that the CA appeared self-centered [13] or irritating [20], especially when the user felt misunderstood [21]. Concerns related to the precision, trustworthiness, and privacy of CAs have been raised as potential obstacles to user engagement and acceptance [22]. Additionally, a growing body of research has begun to unpack MHCA risks, from synthesized case studies of harm [23,24] to empirical studies on harms in CA interactions [25-27] and theoretical exploration of MHCAs’ intrinsic risks [4,28,29]. Together, these accounts suggest that while CAs may offer scalable mental health support, they also introduce new forms of vulnerability, which demand thoughtful, safe design, and robust ethical oversight.

Several frameworks exist to guide the implementation and evaluation of health care technologies, including the Proctor model [30], the Consolidated Framework for Implementation Research [31], and the RE-AIM (Reach, Effectiveness, Adoption, Implementation, and Maintenance) framework [32]. While useful for general health care interventions, these models do not specifically address the unique challenges of designing conversational AI tools [33] such as MHCAs. In human-computer interaction (HCI) and AI literature, more targeted guidelines for human-AI interaction have been proposed. For example, Amershi et al [34] developed 18 design guidelines derived from literature and industry practice, refined through heuristic evaluation and expert review, providing general guidance for AI-infused products. Similarly, Yang and Aurisicchio [35] conducted interviews to construct 10 guidelines for voice assistants, emphasizing competence, autonomy, and relatedness, and recommending features such as transparent system capabilities, socially appropriate conversation design, customization, and data control. In terms of adapting clinical concepts for therapeutic CAs, Moore et al [27] evaluated MHCAs from the lens of basic, crucial prerequisites for therapeutic professionals’ conduct, while Song et al [36] used therapeutic alignment to interpret MHCA users’ experiences. Despite these contributions, there remains a lack of guidance and consistency on equitable, inclusive, and trauma-informed design specifically for CAs in mental health contexts.

Trauma-informed care (TIC) is a strength-based framework for service delivery that emphasizes understanding and responding to the widespread, disempowering effects of trauma [37]. It involves acknowledging the impact of trauma and intentionally responding in ways that support safety and avoid retraumatization [38]. Many individuals experience traumatic events throughout their lives, regardless of their diagnoses or presenting conditions [39], making trauma-informed approaches foundational in mental health care [40]. Trauma-informed approaches aim to maximize physical, psychological, and emotional safety in all health care interactions [37] and not only those explicitly focused on trauma while also fostering opportunities for empowerment, control, and healing through safe, collaborative patient-clinician relationships [41]. Originally proposed by the Substance Abuse and Mental Health Services Administration (SAMHSA), the leading federal agency addressing mental health services in the United States, the TIC framework includes the following 6 key principles: safety; trustworthiness and transparency; peer support; collaboration and mutuality; empowerment, voice, and choice; and cultural, historical, and gender issues [38].

While the TIC framework was initially developed to enhance therapeutic experiences and outcomes in individual psychotherapy and inform organizational policies, its application to technology design is increasingly recognized. In recent years, TIC concepts have been extended to domains such as telehealth and computing [42-44]. Trauma-informed telehealth research provides strategies for clinicians to promote safety, trust, and support during virtual visits [42], while trauma-informed computing emphasizes a sustained commitment to designing digital systems that acknowledge trauma and its effects [43,44]. These approaches offer guidance for creating online environments that are trauma-sensitive and prioritize user safety, agency, and emotional well-being [43-45].

Given that digital technologies can inadvertently trigger or amplify trauma [43], establishing design practices that minimize technology-facilitated harm and retraumatization is essential. As the TIC framework aims to enhance therapeutic experiences and outcomes across individual psychotherapy and organizational policy [46], extending its application to technology design, particularly in the context of AI-based MHCAs, is both relevant and necessary [43]. Applying the TIC framework to CA design has the potential to improve their effectiveness as mental health interventions while addressing known risks, including user codependence [23,47], limited capacity to interpret complex emotional or nonverbal cues [28], and potentially harmful responses to sensitive disclosures [23,25,48]. Consistent with these concerns, systematic reviews of mental health CAs have identified user safety [1,9,10] and trust within the user-CA relationship [4,29,47] as critical and ongoing priorities for current and future research.

While trauma-informed ideas have been discussed across various areas of computing, to date, there has been no systematic effort to apply them to MHCAs. This gap is noteworthy, given the high prevalence of trauma among individuals seeking support through digital mental health tools [43]. The absence of a trauma-specific evaluative framework limits our ability to assess whether current MHCA designs adequately promote safety; trustworthiness and transparency; collaboration and mutuality; empowerment, voice, and choice; sensitivity to cultural, historic, and gender issues; and peer support to protect end users with trauma histories. Although previous literature reviews have extensively examined the efficacy, usability, and safety of MHCAs [1,9,10,21,49-51], it remains unclear how, or to what extent, existing interventions align with or operationalize TIC principles.

We selected SAMHSA’s TIC framework as the guiding lens for this review, as it provides a robust and translatable [42-44] foundation for evaluating trauma-informed design in digital contexts. By applying this framework, this review aimed to bridge a disciplinary gap between clinical care and technology design to provide a structured, trauma-aware evaluation of MHCA research and design to date. Accordingly, this scoping review maps how TIC principles are reflected, explicitly or implicitly, within existing AI-based MHCA research and identifies areas where trauma-informed approaches remain underused but could improve user experience and clinical outcomes. Our guiding research questions are as follows:

1. Which TIC principles are most frequently explored or integrated in the evaluation of MHCAs, and how are they operationalized?
2. What key design considerations and recommendations are proposed in the literature for integrating TIC principles into MHCAs?
3. Are there significant gaps in the literature regarding the application of TIC principles in CA technologies for mental health? If so, what areas require further exploration?

## Methods

### Eligibility Criteria

We followed the PRISMA-ScR (Preferred Reporting Items for Systematic Reviews and Meta-Analyses extension for Scoping Reviews) checklist ( to ensure the reliability of our results [52]. To meet the eligibility criteria, the studies had to (1) evaluate an MHCA for efficacy (either in symptom management or user experience), (2) center participants who were the main users of the MHCA, (3) only look at MHCAs that were independent mobile apps or web-based apps with no human involvement, (4) be published in peer-reviewed journals or conference proceedings in English between 2000 and 2024, and, finally, (5) include explicit or implicit references to principles from SAMHSA’s TIC framework [38,46,53]. We did not register a review protocol prior to conducting the study.

We focused on mobile- and web-based MHCAs because these platforms represent the most accessible and widely used forms of digital mental health interventions in everyday contexts. This narrower scope aligns with prior reviews examining CAs for mental health [1,54], allowing for a more nuanced understanding of how trauma-informed design choices are implemented in the tools with which people most frequently engage. We did not include “social robots” or “embodied agents” as search terms but did exclude papers about physical robots, as their design architectures, usage contexts, and modalities differ from app- and web-based chatbots. Some papers used the term “embodiment” to refer to visual depictions or avatars of text- or audio-based CAs; we included these papers.

Articles were excluded if the MHCA was embedded in a preexisting communication platform (eg, Facebook Messenger), ensuring that the system was purpose-built for mental health intervention rather than serving as an ancillary feature. Extended abstracts and posters were excluded because they typically present preliminary or early-stage work that lacks the methodological and analytical depth needed to assess TIC principles. In contrast, we included both commercially available and research prototypes, provided they presented complete or well-documented evaluations relevant to TIC principles. This approach ensured that the review captured both mature systems currently in use and innovative prototypes that may inform future trauma-informed design in mental health technologies (Textbox 1).

Textbox 1. **Eligibility criteria for scoping review.**

**Inclusion criteria:**

- Study focus: evaluation of mental health conversational agent (MHCA) for efficacy in symptom management or user experience
- Trauma-informed care (TIC): mentions at least one principle from TIC framework
- User interaction: primary users using MHCA for a mental health–related concern
- Conversational agent (CA) type: independent mobile or web app
- CA design: has no human involvement
- Study type: randomized controlled trial, quasi-experimental trials, experimental designs, user studies, pilot studies, observational research, and between-subject studies
- Article type: peer-reviewed articles, journals, and conference proceedings
- Language: English
- Year: studies published between 2000 and 2024

**Exclusion criteria:**

- Study focus: not evaluating an MHCA
- TIC: does not mention at least one principle from TIC framework
- User interaction: CA is not meant for mental health or primary users are not using it for a mental health–related concern
- CA type: not an independent mobile or web app; a physical robot
- CA design: has human involvement
- Study type: Wizard of Oz studies, systematic or scoping reviews, analyses of user reviews
- Article type: abstracts, extended abstracts, dissertations, editorials, position statements
- Language: other languages than English
- Year: studies not published between 2000 and 2024

To identify relevant articles, we searched Google Scholar, the Association for Computing Machinery (ACM) Digital Library, and PubMed in August 2024 for publications from 2000 to 2024. Our search terms for all 3 databases were as follows: (“mental disorder*” OR “mental health” OR “mood disorder*” OR autism OR “depression” OR “anxiety” OR phobia OR bipolar OR schizophrenia OR affective disorder OR psychosis OR psychotic disorder OR obsessive compulsive disorder OR panic disorder OR post-traumatic stress disorder OR substance abuse OR eating disorder) AND (“conversational agent*” OR “artificial intelligence*” OR “conversational AI*” OR “conversational bot*” OR “CAI*” OR “conversational system*” OR “conversational interface*” OR “smart-bot *” OR “virtual agent *” OR “virtual coach *” OR “avatar*” or “chatbot*” OR “chat bot*” OR “chatterbot*"). These search terms were based on previous studies on MHCAs [1,55-57]. Although suicidality is a critical aspect of mental health, we did not include “suicide” or “suicidality” as explicit search terms. This decision followed prior scoping reviews on MHCAs [1,54], which focused on common diagnostic conditions, such as depression, anxiety, and post-traumatic stress disorder, domains where suicidality frequently co-occurs.

Following the initial retrieval of articles from the databases (N=25,857; ACM Digital Library: n=17,084, 66.07%; Google Scholar: n=220, 0.85%; and PubMed: n=8553, 33.08%), 4 authors (FFN, RP, ER, and KV) independently and manually screened the titles and abstracts to assess eligibility based on predefined criteria. The 4 authors divided the databases year-wise (ie, each was assigned a 5-y span). After this initial screening based on titles and abstracts, out of 25,857, a total of 99 (0.4%) articles were retained. Next, all 5 authors conducted relevancy coding by reviewing the full texts of these 99 articles based on the inclusion criteria. Relevancy coding was documented in Microsoft Excel. Discrepancies were discussed collectively and resolved through consensus. Of the 99 full-text articles, 81 (81.82%) were excluded—1 due to duplication and 80 for not meeting the inclusion criteria (user interaction: 37/80, 46.25%; article type: 5/80, 6.25%; CA type: 20/80, 25%; CA design: 2/80, 2.5%; study type: n=13/80, 16.25%; and no TIC elements: 3/80, 3.75%), resulting in 18 (18/99, 18.18%) studies. To broaden the scope of the review, the authors searched through these 18 papers’ citations to identify additional papers. A total of 62 potentially relevant references were manually screened. By consensus, 20 additional studies were included, resulting in a final sample of 38 papers for review. During screening, we used a broad, inclusive definition of references to the TIC framework that allowed us to explore how its core concepts were interpreted and operationalized across contexts without restricting the search to predefined terminology.

### Data Extraction and Analysis

Five authors (FFN, FK, RP, ER, and KV) coded the data in Excel for a broad array of information on each publication in the corpus; publications were randomly assigned among authors. They extracted basic information per publication, including study methodology and outcomes and characteristics of the CA intervention, aligned with the PRISMA-ScR structure and previous studies [1]. Apart from the initial characterization of the studies, the analysis followed an abductive, theory-informed approach [58], combining deductive sensitization to SAMHSA’s TIC principles with openness to inductively derived subthemes reflecting how these principles were operationalized across studies. As none of the papers explicitly referenced or applied SAMHSA’s TIC framework, identifying the 6 TIC principles required careful interpretive analysis. Descriptive and interpretive analyses of all extracted TIC-related data were conducted by the first and last authors, who iteratively revisited the data throughout the writing process to ensure that nuances in how TIC-related ideas were represented were accurately identified and not overlooked. To ensure methodological rigor and reduce subjectivity, the authors used a multistep, consensus-driven coding process. Discrepancies were systematically discussed and resolved through consensus, followed by iterative recoding to refine consistency and reliability. While this approach strengthened the credibility and reproducibility of our thematic interpretations, no qualitative work is neutral, and all interpretation was shaped by authors’ professional experiences and positionalities, as detailed in the next section.

### Author Positionality

Embedded within a large health system, the interdisciplinary research team includes members with expertise in HCI (FK and FFN), social computing (FK and FFN), public and mental health domain (KV, RP, and FFN), health informatics (FFN, RP, and FK), and user experience research and design (ER, FFN, and FK). The team’s work is informed by ongoing collaboration with clinical providers and experience in engaging with mental health–related data, digital interventions, and sociotechnical systems in health care settings.

Clinical input from a licensed provider (a clinical psychologist from the same health system with expertise in trauma and TIC) was sought during the design of the study. Some team members have participated in trauma-informed technology design training within and outside of their health institution. However, our perspective is primarily grounded in applied informatics research and health care practice, shaped by close collaboration with clinicians and work in mental health settings. The team also includes researchers from both Western and non-Western backgrounds.

### TIC Framework

The TIC framework, as defined by SAMHSA, provides a foundational approach for recognizing and responding to the impact of trauma across health care and other service systems [38]. Within this framework, trauma is defined as a combination of experiences that an individual perceives as harmful or life-threatening and the lasting adverse effects of those experiences on the individual’s functioning and well-being [38,46]. Rooted in research and expert consensus, the TIC framework is guided by six interconnected principles: (1) *safety*, ensuring physical and emotional safety in environments and interpersonal interactions; (2) *trustworthiness and transparency*, building trust through transparent, consistent, respectful, and fair communication and decision-making; (3) *collaboration and mutuality*, promoting partnership and reducing power imbalances between individuals, whether staff or clients; (4) *empowerment, voice, and choice*, recognizing and strengthening individuals’ existing capacities, voices, and experiences; (5) *peer support*, valuing and incorporating the perspectives and support of those with lived experiences of trauma; and (6) *cultural, historical, and gender issues*, being responsive to cultural, racial, historical, and gender-based contexts that shape individuals’ experiences [38,53]. These principles are designed to promote environments that acknowledge trauma’s pervasive effects, recognize its signs and symptoms, integrate this understanding into practice, and actively seek to prevent retraumatization [46]. We apply the TIC framework as a critical lens for evaluating mental health chatbots (MHCAs), as these digital tools often interact with users during moments of psychological vulnerability, and their design choices can either mitigate or exacerbate distress.

## Results

### Overview of Included Publications’ Metadata (n=38)

**Figure 1.**
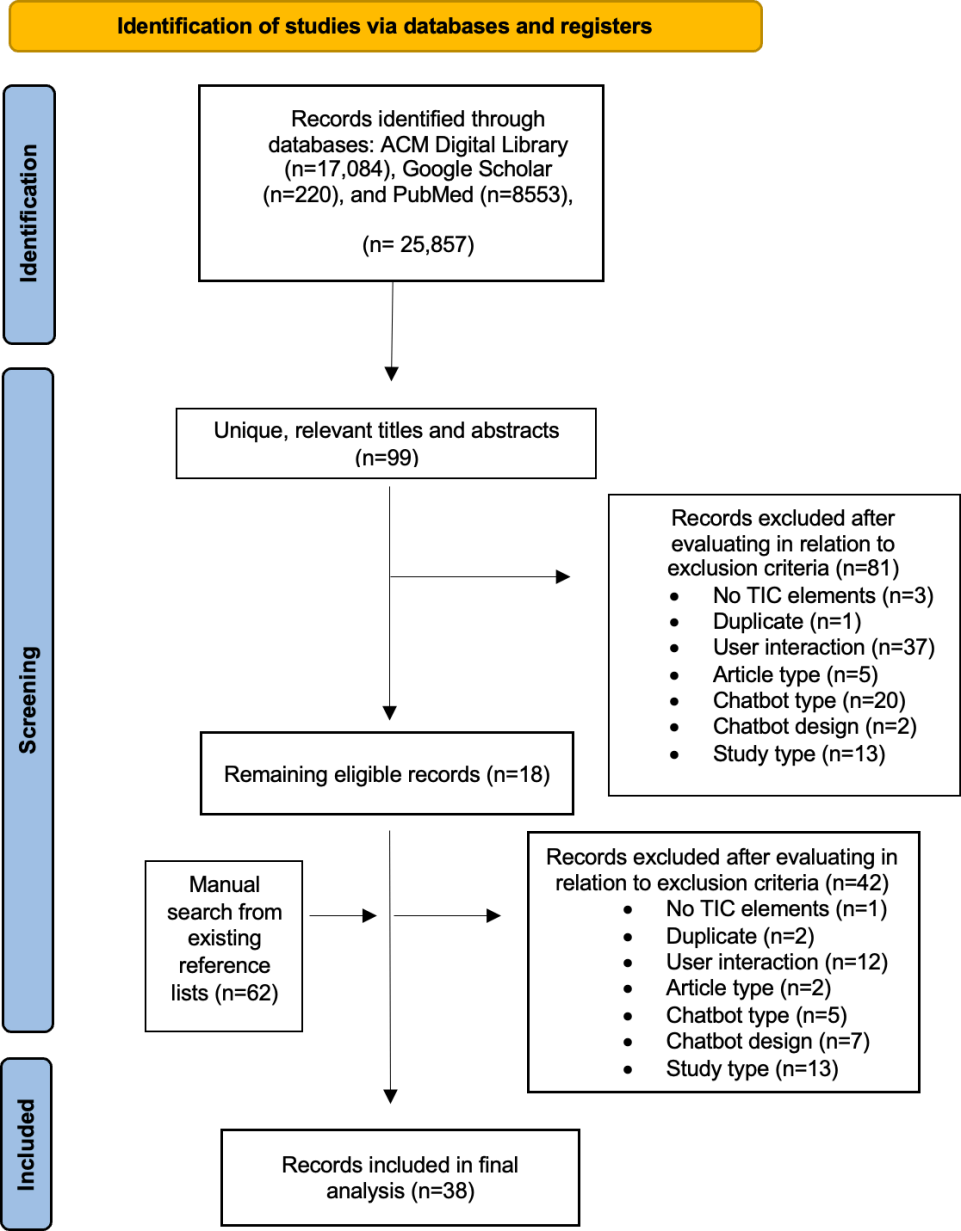
PRISMA (Preferred Reporting Items for Systematic Reviews and Meta-Analyses) Flowchart outlining the identification and screening of publications for scoping review. TIC: trauma-informed care.

Checklist 1 represents the PRISMA-ScR checklist we followed. The study selection process is summarized in Figure 1. A total of 18 (47.4%) publications were identified through the initial database search (PubMed: 9/38, 23.7% [12,13,59-65]; the ACM digital library: 7/38, 18.4% [66-72]; and Google Scholar: 2/38, 5.2% [73,74]; Tables1  2). The remaining 20 (52.6%) publications were found as citations of these 18 sources [75-94]. Overall, 30 out of 38 (78.9%) were journal articles, with the most frequent venues being the *Journal of Medical Internet Research* and affiliated journals (15/30, 50% articles), followed by *Frontiers in Digital Health* (4/30, 13.33% articles); 8 out of 38 (21.1%) were conference proceedings. Most (28/38, 73.7%) studies were published in 2020 or later, reflecting a recent surge in the domain. The United States was the most common study location (13/38, 34.2% studies), whereas 12 (31.6%) out of 38 articles involved users based in various European countries. While most studies’ participants were from a single country, 4 (10.5%) studies included participants from multiple countries [66,76,83,84], signaling some movement toward globally inclusive development.

**Table 1.** Basic characteristics of included publications (n=38).

Parameters and characteristics	Studies^a^
Publication metadata, n (%)	
Study design^b^	
Randomized trial	18 (47.4)
Other experimental study types	5 (13.2)
User study	141 (28.9)
Pilot study	67 (18.4)
Survival analysis	1 (2.6)
Type of publication	
Journal article	30 (78.9)
Conference proceeding	8 (21.1)
Publication source	
Google Scholar	2 (5.3)
PubMed	9 (23.7)
ACM^c^ Digital Library	7 (18.4)
Manual search	20 (52.6)
Study location^d^	
United States	13 (34.2)
China	4 (10.5)
Ireland	3 (7.9)
United Kingdom	3 (7.9)
Switzerland	1 (2.6)
Australia	2 (5.3)
Norway	1 (2.6)
Brazil	1 (2.6)
Argentina	1 (2.6)
Sweden	2 (5.3)
New Zealand	1 (2.6)
South Korea	1 (2.6)
Philippines	1 (2.6)
France	1 (2.6)
Finland	1 (2.6)
Scotland	1 (2.6)
Japan	1 (2.6)
The Netherlands	1 (2.6)
Belgium	1 (2.6)
Not available	3 (7.9)
Year of publication	
Prior to 2020	10 (26.3)
2020-2022	21 (55.3)
2023-2024	7 (18.4)
Sample characteristics	
Sample size, n (%)	
≤50	11 (28.9)
51-100	6 (15.8)
101-200	12 (31.6)
201-499	4 (10.5)
≥500	5 (13.2)
Age (y)^e^	
Mean (range)	32.52 (17‐69.2)
Sex (% male)^f^	
Mean (range)	34.92 (0‐82)
Recruitment setting, n (%)	
Clinical	7 (18.4)
Nonclinical	29 (76.3)
Clinical and nonclinical	2 (5.3)

a Percentages were rounded and may not sum to 100.

b Numbers do not add up as some studies used more than one methodology.

c ACM: the Association for Computing Machinery.

d Numbers do not add up as some studies took place in 2 or more countries.

e Mean age was reported in 25 studies.

f Sex percentages were reported in 32 studies.

A total of 18 (47.4%) studies included in this scoping review were randomized trials (randomized controlled trials: n=17, 94.4%), with 4 (22.2%) identified as preliminary or pilot studies. Sample sizes for randomized controlled trials ranged from 30 to 700 (mean 140.35, SD 153.99; median 107, IQR 58-148). Additionally, 5 (13.2%) studies used other experimental designs, including between-subject studies [66,76], single-arm pre-post intervention studies [74,83], and nonrandomized prospective studies [94]. Eleven (28.9%) publications were based on user studies and included mixed methods (n=6, 54.5%) [13,67,73,78,79,82], qualitative approaches (n=3, 27.3%) [60,68,77], and quantitative approaches (n=2, 18.2%) [70,75]. Observational analyses of commercial app data (5/38, 13.2%) [13,65,78,88,89] featured the largest sample sizes of all study types (mean 2334.8, SD 1775.8; median 2194, IQR 667-4073). Finally, 1 (2.6%) study comprised a quantitative survival analysis using data originally collected from a separate clinical trial [62].

Overall, participant samples in the reviewed studies skewed younger (mean age 32.52, SD 14.6 y) and female (male participants: mean 34.92%, SD 18.64%). Twenty-nine (76.3%) publications involved only nonclinical samples, whereas 7 (18.4%) included clinical samples. Two (5.2%) studies recruited both clinical and nonclinical samples for comparison [67,83]. Depression and anxiety were the most frequently addressed concerns across the studies included [12,13,59,61,62,67,70,78,80,83,86]. Summaries of publications can be found in Table 1; more information is available in Multimedia Appendix 1.

### Overview of MHCA Interventions Reported in the Included Studies (n=28)

Most MHCAs were reported in a single publication, although a few appeared in multiple studies (eg, Woebot was included in 6 studies, and Wysa was included in 5 studies). While most publications evaluated a single MHCA, some did multiple MHCAs [73,90,93]. Thus, 28 distinct MHCAs were represented across the corpus. Seventeen (60.7%) of 28 MHCAs were described as prototypes at the time of the study, and 9 (32.1%) were commercially available. However, close to half (17/38, 44.7%) of the publications in the corpus focused on commercial MHCAs. The most popular MHCAs were Woebot [12,68,70,73,74,87] and Wysa [13,62,73,78,88]. Text (including emojis) or multiple-choice options were the most common input and output modalities, with 13 (13/28, 46.4%) MHCAs including other modalities such as audio or video. Sixteen (16/28, 57.1%) MHCAs used rule-based functionality for conversation logic, 1 (1/28, 3.6%) was fully generative AI, and 9 (9/28, 32.1%) incorporated both. Eighteen (18/28, 64.3%) MHCAs were described to have a visual avatar (eg, realistic animated face [84] or nonhuman abstract character [77]). Fourteen (14/28, 50%) MHCAs were described as having a form of crisis intervention. While other MHCAs could have this feature, it was not directly mentioned in the articles.

One component of design frequently absent from included articles was the extent to which the MHCA versus the user guided the interaction, as well as examples of representative interactions. Out of 28 MHCAs included in this study, 26 (92.9%) delivered interventions to improve mental health; one (3.6%) focused on diagnosis [82], and another (3.6%) focused on hospital discharge counseling [80]. Mental health improvement interventions were most often based on CBT (14/26, 53.8%), psychoeducation (8/26, 30.8%), mindfulness (4/26, 15.4%), positive psychology (3/26, 11.5%), motivational interviewing (2/26, 7.7%), self-care or self-help (3/26, 11.5%), acceptance and commitment therapy (2/26, 7.7%), gratitude (2/26, 7.7%), and mood tracking (2/26, 7.7%). Eleven (42.3%) of 26 CAs that provided mental health interventions were multimodal, involving 2 or more different approaches. Summaries of MHCA interventions are presented in Table 2, with more information on MHCAs provided in Multimedia Appendix 2.

**Table 2.** Characteristics of mental health conversation agent (MHCA) intervention (n=28).

Parameters and characteristics	Chatbots, n (%)
Purpose	
Mental health intervention	26 (92.8)
Diagnosis	1 (3.6)
Hospital discharge counseling	1 (3.6)
Status	
Commercial	9 (32.1)
Prototype	17 (60.7)
Not available	2 (7.1)
Response generation	
Rule based	16 (57.1)
Artificial intelligence	1 (3.6)
Hybrid	9 (32.1)
Unclear from paper text	2 (5.3)
Input and output modality^a^	
Text was sole input and output modality	3 (10.7)
Text and multiple choice were sole input options	13 (46.4)
Multiple choice was sole input option	5 (17.9)
Included emojis as input or output	4 (14.3)
Include audio or voice as input or output	8 (28.6)
Included infographics or images as input or output	7 (25.0)
Included video as input or output	3 (10.7)
Targeted disorder^b^	
General mental well-being	3 (10.7)
Depression and/or anxiety	10 (35.7)
Substance abuse disorders	1 (3.6)
Emotional distress or stress	6 (21.4)
Eating disorders	2 (7.1)
Schizophrenia	1 (3.6)
Chronic pain	1 (3.6)
Panic disorder	1 (3.6)
Posttraumatic stress disorder	1 (3.6)
Flexible or user-determined targeting	2 (7.1)
No specific targeted disorder	7 (25)

a Numbers do not add up as several MHCAs had more than one input or output modality.

b Numbers do not add up as several MHCAs target more than one health condition.

### Exploration of TIC Principles in Included Publications

#### Overview

Although no publications cited the TIC framework, we explored both explicit and implicit references to its principles [38,46,53]. This approach allowed us to capture trauma-informed practices that may be present but not formally acknowledged in the design and evaluation of the MHCAs, providing a more holistic picture. This echoes the scoping review by Eggleston et al [95], which also found that while digital interventions neither used the term “trauma informed” in their described design processes nor cited SAMHSA, the authors could extract analyzable allusions to TIC principles. It is also important to note that SAMHSA is an American organization. Studies conducted outside the United States may be less likely to align with this framework, as trauma-informed practices can vary across international contexts.

We classified *explicit* references as instances where papers focused on one or more TIC principles using the exact principle names (eg, safety, trust and trustworthiness, and transparency) (Table 3). *Implicit* references to TIC principles reflected discussions of concepts that aligned with or alluded to TIC principles but were not specifically named (Table 4). As the purpose of this review was to evaluate the presence of TIC-aligned ideas across diverse literature, identifying implicit references required careful interpretation. To justify these determinations and limit subjective bias, we cross-checked each potential implicit reference against SAMHSA source texts [37,38,46,53], considered how the concept functioned within the paper’s stated aims and objectives, and engaged in iterative team discussions to reach consensus. The sections that follow outline how these explicit and implicit references were expressed, implemented, and measured.

**Table 3.** Explicit references to trauma-informed care (TIC) principles in included publications, including the name of the principle, the mental health conversational agent (MHCA) and publications that included that principle, and how the TIC-related consideration was measured or included.

TIC principle and subprinciple	MHCA	Implementation
Safety (n=8)		
Safety	Emohaa [59]	Intervention design
Safety	Woebot [73]	Intervention design
Safety	3MR_2 [75]	Intervention design and introduction
Safety	Carebot [76]	Discussion
Perceived safety	KIT [77]	Finding from qualitative user study
Perceived safety	Botstar [66]	Godspeed-V, introduction, and discussion
Perceived safety	Wysa [78]	Discussion and intervention design
Perceived safety	ChatPal [60]	Findings of supplementary qualitative user study and discussion
Trustworthiness and transparency (n=18)		
Trust	Wysa [78]	Definition of therapeutic alliance, associated with WAI-SR^a^ scale
Trust	Laura [79]	Scale response satisfaction questionnaire, introduction, intervention design, and finding
Trust	Elizabeth [80]	Definition of therapeutic alliance, associated with WAI-SR scale
Trust	ChatPal [67]	Scale response questionnaire
Trust	Woebot [68]	Finding from qualitative user study, introduction and discussion
Trust	EMMA [81]	Introduction
Trust	ChatPal [60]	Finding from qualitative user study and discussion
Trust	Philobot [69]	Discussion
Trust	Carebot [76]	Trust in Automation scale, findings of supplementary qualitative user study, and introduction
Trust	Woebot [70]	Findings of supplementary qualitative user study and discussion
Trust	Unnamed [82]	Associated with Acceptability E-Scale and discussion
Trust	ChatPal [83]	Findings of supplementary qualitative user study
Trust	User-chosen name [71]	Discussion
Trust	Botstar [66]	Multi-Dimensional Measure of Trust, introduction, and discussion
Trust	XiaoE [61]	Item in Working Alliance Questionnaire
Trust	Bella [84]	Items in Friendship Questionnaire
Trust and transparency	ChatPal [67]	Discussion
Transparency	Carebot [76]	Item in scale response trust questionnaire and discussion
Transparency	Woebot [73]	Finding from qualitative user study
Transparency	SELMA [85]	Intervention design
Transparency	ChatPal [83]	Finding of supplementary qualitative analysis of user study
Transparency	User-chosen name [71]	Finding and discussion
Collaboration and mutuality (n=11)		
Collaboration	Wysa [78]	Item in WAI-SR and definition of therapeutic alliance
Collaboration	Elizabeth [80]	Item in WAI-SR
Collaboration	SELMA [85]	Item in WAI-SR
Collaboration	XiaoNan [86]	Item in WAI-SR
Collaboration	Woebot-SUDS [87]	Item in WAI-SR
Collaboration	User-chosen name [71]	Item in WAI-SR
Collaboration	Woebot-SUDs [74]	Item in WAI-SR and definition of therapeutic alliance
Collaboration	XiaoE [61]	Introduction
Collaboration	Wysa [62]	Introduction
Collaboration	Multiple names [72]	Introduction
Collaboration	Woebot [70]	Discussion
Peer support (1)		
Peer support	Woebot [73]	Introduction and findings of qualitative user study
Empowerment, voice, and choice (n=3)		
Empowerment	KIT [77]	Finding from qualitative user study
Empowerment	SELMA [85]	Intervention design
Empowerment	ChatPal [60]	Finding from qualitative user study
Sensitivity to cultural, historical, and gender issues (n=2)		
Sensitivity to gender issues	KIT [77]	Finding from qualitative user study
Sensitivity to cultural issues	ChatPal [60]	Introduction

a WAI-SR: Working Alliance Inventory–Short Revised.

**Table 4.** Summary of implicitly trauma-informed care (TIC)–related concepts in the corpus, including the concept name, how many publications included it, and where these concepts were found.

Related TIC principle and implicit TIC concept	Location of implicit TIC concept (n publications)				
	Qualitative findings, n (%)^a^	Quantitative findings, n (%)	System design or method, n (%)	Discussion point, n (%)	Other, n (%)
Safety (n=32)					
Mental health crisis–related content option (n=4)	0 (0)	0 (0)	3 (75)	1 (25)	0 (0)
Crisis or MHCA^b^ failure detection and real-life services or hotlines provision (n=10)	0 (0)	0 (0)	9 (90)	1 (10)	0 (0)
Digital safety–related concepts (anonymity, integrity, password protection, security, and privacy; n=13)	2 (15.4)	1 (7.7)	4 (30.8)	4 (30.8)	5 (38.5)
Input handling for safety (limiting input options and ability to handle unexpected input; n=7)	2 (28.6)	0 (0)	3 (42.9)	2 (28.6)	0 (0)
Enable self-disclosure (n=12)	5 (41.7)	2 (16.7)	0 (0)	4 (33.3)	4 (33.3)
Positive emotional and psychological experience of use (eg, empathy, validation, nonjudgmental, and warmth; n=26)	11 (42.3)	7 (26.9)	9 (34.6)	10 (38.5)	1 (3.8)
24/7 availability (n=4)	2 (50)	1 (25)	0 (0)	1 (25)	1 (25)
Trustworthiness and transparency (n=29)					
Clear instructions and communication (n=14)	7 (50)	1 (7.1)	5 (35.7)	3 (21.4)	0 (0)
Providing information about MHCA (n=7)	1 (14.3)	0 (0)	5 (71.4)	1 (14.3)	0 (0)
Clearly nonhuman (n=5)	1 (20)	0 (0)	3 (60)	1 (20)	0 (0)
Reliability, accuracy, consistency in design, performance, and intervention (n=20)	6 (30)	13 (65)	1 (5)	5 (25)	0 (0)
Nonrepetitive content (n=8)	6 (75)	0 (0)	0 (0)	2 (25)	0 (0)
Collaboration and mutuality (n=26)					
Bond, rapport, relationship, and therapeutic alliance (n=17)	5 (29.4)	11 (64.7)	3 (17.6)	8 (47.1)	3 (17.6)
Mutual respect (n=9)	0 (0)	9 (100)	0 (0)	0 (0)	0 (0)
Goal setting, organization, problem resolution, and accountability (n=16)	4 (25)	9 (56.3)	5 (31.25)	1 (6.25)	0 (0)
Reciprocal, personal communication, and active listening (n=6)	4 (66.7)	0 (0)	1 (16.7)	4 (66.7)	0 (0)
Perception of receiving social support (n=2)	2 (100)	0 (0)	0 (0)	1 (50)	1 (50)
Peer support (n=3)					
Encourages seeking out real-life social support (n=3)	1 (33.3)	0 (0)	2 (66.7)	0 (0)	0 (0)
Empowerment, voice, and choice (n=35)					
Customization, personalization, and flexibility (n=13)	6 (46.2)	2 (15.4)	3 (23.1)	8 (61.5)	1 (7.7)
User control over settings (n=19)	10 (52.6)	1 (5.3)	13 (68.4)	6 (31.6)	0 (0)
Access to or visualizations of past user activity (n=9)	5 (55.6)	0 (0)	6 (66.7)	0 (0)	0 (0)
User autonomy, confidence, motivation, and self-efficacy (n=17)	2 (11.8)	13 (76.5)	2 (11.8)	2 (11.8)	1 (5.9)
Sensitivity to cultural, historical, and gender background (n=25)					
Accessibility (n=16)	3 (18.8)	10 (62.5)	0 (0)	3 (18.8)	2 (12.5)
Usable to diverse users (n=4)	2 (50)	1 (25)	1 (25)	1 (25)	0 (0)
Sensitivity to user characteristics (eg, race and health literacy; n=4)	1 (25)	2 (50)	1 (25)	3 (75)	1 (25)
Sensitivity to user concerns and symptoms (eg, chronic pain and schizophrenia; n=4)	4 (100)	3 (75)	4 (100)	3 (75)	0 (0)
Sensitivity to user language or nationality (n=3)	1 (33.3)	0 (0)	3 (100)	2 (66.7)	0 (0)

a Percentages are calculated using the number of publications addressing each TIC concept as the denominator. A publication may address a given concept across multiple manuscript sections; therefore, percentages may not sum to 100%.

b MHCA: mental health conversational agent.

#### Explicit References to TIC Principles

One or more principles from the TIC framework were explicitly mentioned across 26 publications (26/38, 68.4% of publications; Table 3). All TIC principles were referenced by name (although, again, not *citing* the TIC framework) at least once in all publications. Most publications that named TIC principles did not provide working definitions of them (except [76], who defined “trust”), making it difficult to identify how they were operationalized. Studies that named TIC principles more frequently involved prototypes (17/26, 65.4%). The MHCAs mentioned most often in this subsection of the corpus were Woebot [68,70,73], ChatPal [60,67,83], and Wysa [62,78].

Half (50%) of the 8 studies that explicitly referenced *safety* addressed felt or perceived safety (ie, as an end user experience) that was tied to feeling that an MHCA would not be triggering [77], knew right from wrong (ie, moral agency) [66], successfully developed a therapeutic alliance [78], or offered true anonymity [60]. Similarly, safety was emphasized alongside confidentiality as a discussion point by Moilanen et al [76]. While no included publications used the phrases “emotional safety” or “psychological safety” [38,53], Wester et al [66] measured perceived safety by assessing whether users felt calm, anxious, or neutral while interacting with the system (using a Godspeed questionnaire subscale). Similarly, an additional 4 publications did not use the phrase “physical safety,” but related safety to intervention design features such as detecting and intervening when suicidal ideation was detected [59], helping users create a safety plan [73], ensuring provided resources did not promote harmful advice [76], and including human-in-the-loop monitoring for worsening symptoms by clinicians [75].

Numerous studies identified trust or *trustworthiness* as important and a determinant of user acceptability [60,66,67,69,70,76,81,82] and a component of meaningful emotional support [68,70]. Three (16.7%) studies out of them linked trustworthiness to data privacy, anonymity, confidentiality, and secure data storage [60,67,68]. References to *transparency* usually described it as a part of trustworthiness [71,76,83] (eg, participants said they would “be more likely to trust the chatbot if it was transparent about its purpose and what would happen to user data at the very start” [83]). Moilanen et al [76] were the only authors who offered a definition of trustworthiness or transparency, describing trust as an attitude that a CA will help achieve an individual’s goals in situations marked by uncertainty and vulnerability. To assess trust, reviewed studies used both standardized [66,76] and custom [67] survey instruments. For example, the Trust in Automation scale evaluated whether the prototype, Carebot, was perceived as reliable and trustworthy or, conversely, as deceptive and harmful [76]. Another study developed a bespoke survey to understand users’ trust in ChatPal [67]. Although not trustworthiness surveys per se, the Working Alliance Inventory–Short Revised (WAI-SR), Working Alliance Questionnaire, and Friendship Questionnaire included items about trust [61,78,80,84], recognizing a conceptual overlap between trust and therapeutic alliance [78].

*Collaboration* was similarly frequently mentioned by name in the corpus (11/38, 28.9%, publications). Several papers that explored the therapeutic or working alliance between MHCAs and users engaged directly with collaboration and mutuality through items on different versions of the Working Alliance Inventory (WAI) screening tool (eg, WAI-SR and Working Alliance Inventory, Short form, Dutch version [WAV-12] scale) [71,74,78,80,85-87]. Beatty et al [78] emphasized fostering a therapeutic alliance grounded in collaboration, trust, empathy, and genuineness. Similarly, He et al [61] underscored the value of collaborative MHCA interactions that support co-development of well-being strategies. While these 2 studies addressed collaboration and mutuality from a broader theoretical and design perspective, De Nieva et al [70] highlighted specific user preferences, finding that MHCAs’ frequent failure to understand user input or context diminished trust, rapport, and the overall sense of therapeutic alliance—ultimately weakening perceived collaboration.

Only 3 (7.9%) studies directly engaged with the principle of *empowerment, voice, and choice* in their studies [60,85]. Hauser-Ulrich et al [85], in their system design process, said that CBT approaches can empower users to manage their own mental health, although this mention was relatively casual. In contrast, Kostenius et al [60] explored empowerment in their findings: users of ChatPal described how the MHCA helped them feel more in control and gave them hope that things could improve, suggesting that the experience of empowerment was actively facilitated through tone and functionality. Finally, in one line of user feedback, Beilharz et al [77] noted that getting specific, actionable advice from the MHCA would make them feel empowered.

Of all the studies reviewed, only 2 (5.3%) explicitly acknowledged the importance of designing and evaluating MHCAs with *sensitivity to target users’ gender and cultural background*s [60,77]. Kostenius et al [60] highlighted that designing culturally adapted CAs for minorities can “bridge the gap between language, culture, and professionals’ understanding of [mental health] factors.” They evaluated ChatPal in Sweden and gathered user feedback to assess the multilingual MHCA’s performance in non-English contexts and found that an absence of proper language for mental health concerns in different languages can create trust issues and impact the tool’s overall experience and adoption. Beilharz et al [77], also analyzing user feedback, showed that gender identity can affect how safe users feel while disclosing sensitive information, particularly for those struggling with body image issues; thus, designing MHCAs aligned with users’ gender preferences can facilitate a safer environment. These studies explored the impact of users’ cultural and gender backgrounds via qualitative data and did not include them quantitatively or as discussion points. Explicit mention of any *historical* issues was also missing in the corpus.

While other TIC principles were explored by name to an extent, *peer support* was notably absent: no studies defined or measured peer support. In the sole publication to reference the principle by name, reported qualitative user feedback indicated a desire for more effective integration of group or peer support elements [73]. However, the paper did not provide any discussion or design strategies for how peer support could be implemented.

#### Implicit References to TIC Principles

In addition to instances where TIC principles were explicitly named, all 38 publications incorporated descriptions that *implicitly* aligned with the TIC framework, conveying similar values such as positive emotional and psychological experiences, rapport, reliability, and accessibility. While Table 4 provides a snapshot of those concepts, a more detailed breakdown of implicit TIC-related elements can be found in Multimedia Appendix 3.

#### Implicit References to Safety

Twenty-six (81.3%) out of 32 studies that implicitly referenced safety emphasized creating a *positive emotional and psychological experience* for user-MHCA interactions. They highlighted the importance of empathy, emotional validation, nonjudgmental responses, and warmth in user-MHCA interactions [12,64,84,92], qualities closely aligned with SAMHSA’s definition of emotional and/or psychological safety [38,53]. These elements were often framed as contributing to positive user experiences rather than explicitly as safety mechanisms, and they primarily emerged through qualitative user feedback (11/26, 42.3%, publications) or were integrated within the system’s design (9/26, 34.6%, publications).

*Digital safety* also appeared frequently, with 13 (40.6%) publications referencing anonymity, privacy, security, integrity, or password protection. Although not included in SAMHSA’s original framework, these concerns are included in extensions of safety for digital contexts [43,44]. Anonymity was often framed as enabling self-disclosure [60], while security and integrity were discussed as prerequisites for system trust [76]. Additionally, concepts such as data protection [89,90] and user privacy [59,63] were also advocated in the studies. Digital safety–related constructs were thus entangled with felt psychological safety, trust, and the material protection of users’ sensitive data in MHCA interactions. For 4 (30.8%) out of 13 studies, digital safety–related concepts appeared only in framing sections (ie, the introduction, literature, or conclusion) rather than in evaluated system features [13,61,71,94].

A total of 13 (40.6%) publications discussed mental health crisis–related interventions (either as a *content option* or as *automated detection and services or hotline provision*), which can be interpreted as addressing users’ physical safety (one half of the TIC principle of safety [38]), as crises in these studies were typically defined in terms of suicide or self-harm risk. Four (30.8%) out of these 13 studies embedded *crisis-related content within the system* [64,77,83,86], and 10 (76.9%) studies described *automated detection of crisis language* (eg, self-harm [71] or suicidal ideation [59,91]) or *MHCA failure* (ie, for ChatPal [60,83]), followed by *referrals to external support services* [59,60,63,71,74,76,83,89,91,92]. However, these mechanisms were rarely evaluated empirically.

In addition to these digital, physical, and emotional safety-related concepts, 4 (12.5%) studies noted the value of *24/7 availability,* enabling users to engage whenever support is needed [60,68,71,88]. As noted in a SAMHSA Treatment Improvement Protocol, “other key elements in establishing a safe environment include consistency in client interactions and treatment processes...and dependability” [37]. We conceptualize constant MHCA availability, particularly as a nonhuman digital service, as a form of consistency and dependability, although this idea overlaps considerably with definitions of trustworthiness [38,53], as we discuss next.

#### Implicit References to Trustworthiness and Transparency

Trustworthiness and transparency appeared implicitly across many studies through discussions of system *reliability* and *consistency*, both described in definitions of that TIC principle [38,53]. Twenty (70%) out of 29 publications that implicitly referenced trustworthiness and transparency related concepts emphasized that users valued MHCAs that provided *accurate* information and performed *reliably without technical glitches*. These elements were included as questions in various validated measures, including those related both to usability (eg, System Usability Scale [SUS] [64]) and trust (Trust in Automation scale [76] and Multi-Dimensional Measure of Trust [66]). *Clear communication* (ie, transparency) *about MHCA functions and roles* also appeared in 7 (24.1%) studies, primarily in MHCA design documentation [74,75,79,85,87,89], while a desire for *clear instructions and communication* from the MHCA appeared in 14 (48.3%) publications, often as findings from supplementary qualitative data [59,60,64,70,76,83]. These elements were usually framed as standard usability features, alongside *nonrepetitive MHCA responses*, which were also typically highlighted through supplementary user feedback [12,13,60,61,64] and rarely as a primary study focus or outcome.

#### Implicit References to Collaboration and Mutuality

Several studies assessed constructs such as *therapeutic alliance, working alliance, and interpersonal closeness* between users and CAs, which we identified as proxies for evaluating collaboration and mutuality (and can also be interpreted as trustworthiness and transparency). Collaboration and mutuality, per SAMHSA, include partnering and meaningful sharing of power and decision-making [38]. In the corpus, *therapeutic or working alliance* was defined as a collaborative, emotionally engaged relationship marked by shared goals and commitment to therapeutic tasks [61,78-80,85]. Out of 17 publications that touched on similar concepts, relationships were commonly assessed through quantitative tools (12/17, 70.6%; eg, WAI and its variants [71,72,74,78,80,85-87]), whose items assessed goal agreement, task collaboration, mutual respect, and emotional bond. Other studies highlighted relational behaviors (per SAMHSA, collaboration is a demonstration that “healing happens in relationships” [38]) in supplementary qualitative user feedback and discussion sections, including *active listening* [70], *addressing the user by name* [71,76,84], *remembering past conversations* [59,76], *asking follow-up questions* [66], and *checking in* [70,74].

#### Implicit References to Empowerment, Voice, and Choice

Closely related to the concepts in the previous section, 9 (25.7%) of 35 publications that implicitly referenced empowerment, voice, and choice highlighted benefits of *providing visualizations of or access to users’ past data*, as it enabled progress tracking, allowing users to draw insights and be key decision-makers in their working relationship with the MHCA. *User control over the MHCA* (19/35, 54.3%, publications), although, was the most common implicit expression of empowerment, voice, and choice. In the TIC framework, empowerment, voice, and choice of clients include fostering their resilience, recognizing their strengths, and centering them as in charge of their recovery, as trauma typically involves a loss of autonomy or control [38,53]. Across the corpus, user control was reflected in features such as the ability to opt out of specific functions [62,63,70,81,92] and the provision of diverse input and output modalities that enabled flexible expression and engagement [59,60,64,65,73,77,80,81,83-85,89,94]. These considerations were most frequently articulated in system design descriptions (14/19, 73.7%, publications), but they also emerged in qualitative findings (10/19, 52.6%, publications).

Empowerment, voice, and choice were also expressed implicitly via *customization, personalization, and flexibility* (13/35, 37.1% of publications). While not explicitly named in TIC principle definitions, we interpret these concepts as extensions of user control and choice, as they reflect respect for individual needs embedded in design. Many studies identified greater customization as a direction for future MHCAs, although this was often discussed as a recommendation rather than empirically evaluated [66,72,76,78,86], except for Vossen et al [71]. Personalizability functioned as their primary independent variable; results showed preferences for customization of therapeutic approach, appearance, and conversational style [71].

Thirteen (37.1%) publications included quantitative user experience scales (eg, SUS [64,67,69,75,93], Mobile Application Rating Scale [user version] [73], other satisfaction surveys [12,72,92], and acceptability surveys [61]) that measured what we identified as a felt sense of empowerment, voice, and choice by participants (eg, of *user autonomy, confidence, motivation,* and *self-efficacy*). Specific items of these scales asked users about feeling confident [64,67,69,72,74,75,79,84,87,91,93], motivated [73], self-aware [12,92], and/or able to engage in self-help [61,77] in relation to their interactions with the MHCA.

#### Implicit References to Sensitivity to Cultural, Historical, and Gender Issues

When addressed implicitly, sensitivity to cultural, historical, and gender issues most often appeared as a general call for *accessibility* (16/25, 64% of publications). Within the TIC framework, sensitivity is defined as responsiveness to client characteristics and includes deconstructing stereotypes and biases [38,53]. In the corpus, *accessibility* was operationalized through measures and design to make MHCAs usable for a range of users and needs. Similar to empowerment-related constructs, accessibility was primarily evaluated quantitatively using standardized user experience measures (10/16, 62.5%, publications), including items on the SUS [64,67,69,75,93], the Usability Metric for User Experience-LITE (user version) [61], and the Chatbot Usability Questionnaire (CUQ) [67]. We interpret these measures as capturing whether users experienced the MHCA as responsive, even when cultural, historical, or gender-specific considerations were not foregrounded in detail.

Beyond usability, a smaller subset of studies engaged more directly with how *specific user characteristics* shaped MHCA design or outcomes. These included attention to health literacy [80], visual racial markers [72], nationality [91], language [60,83], and age (eg, teenage users [66]). One New Zealand–based study designed its MHCA’s animated face to appear mixed-race Māori and New Zealand European; while this choice was not formally justified or evaluated, 1) participant reported feeling “represented” by the agent’s appearance [84]. Other studies focused on clinically specific populations, tailoring MHCAs to *symptom profiles*. For example, Bickmore et al [79] designed an MHCA for individuals with schizophrenia to support medication adherence using psychiatric nursing best practices for psychosis, while Meheli et al [88] and Sinha et al [62] examined how chronic pain shaped users’ mental health needs and interactions with MHCAs, informing condition-specific design recommendations.

#### Implicit References to Peer Support

In the 3 (7.9%) publications out of the whole corpus that mentioned seeking support from other people (not necessarily “peers” or other trauma survivors and/or caregivers, per SAMHSA [38]), all discussed design features that allowed MHCA users to *seek out real-life social support*. For 2 (66.7%) out of the 3 studies, these features were embedded in the systems they evaluated: one [79] asked users to provide contact information for a support person, and the other [77] included a “find support groups” feature. Neither study explored the feature in findings or discussion sections. Finally, Balaskas et al [73] mentioned user suggestions for human support incorporated into the MHCA app in their qualitative findings but did not engage with it further.

### TIC-Related Design Considerations and Recommendations

Whereas the previous sections document how TIC principles appeared across MHCA research, this section presents concrete design recommendations. As most reviewed papers did not include formal design recommendation sections, we extracted actionable guidance wherever authors reflected on design implications, including system descriptions, findings, and discussions. As design recommendations can intersect with multiple TIC principles, our classifications are intentionally flexible rather than rigid and should not be interpreted as mutually exclusive. Textbox 2 presents a summary, while Multimedia Appendix 4 breaks overarching themes down by publication, categorizing each reference according to which explicit principle (ie, Table 3) or implicit concept (ie, Table 4) it is connected to.

Textbox 2. **Design recommendations associated with explicitly and implicitly trauma-informed care (TIC)–related elements in corpus. Breakdowns by implicit or explicit element and by publication are in Multimedia Appendix 5.**

Related TIC principle and all related design recommendations

- Safety
  - Provide flexible access options, allowing users to engage anonymously without mandatory registration [60,68,76,78,88] while also offering secure login credentials [89,90] for those who prefer personalized privacy and data protection.
  - Instruct users not to share any personally identifiable information [59].
  - Provide a stigma- and judgment-free [61,66,77,82,84,88] environment for self-disclosure [68,74,82,83].
  - Implement crisis detection through language analysis [59,63,71,74,76,89,91] to provide immediate helpline information [60,63,83,89,91,92] and clearly signal to first-time users that the mental health conversational agent (MHCA) is not a crisis service [74,87] or a replacement for care [85,89].
  - Provide a crisis content module that creates a safety plan [73] or includes helpline and emergency services information [64,77,83,86].
  - Ensure patient safety by introducing a clinician in the loop to check questionnaire scores and directly call users [75].
  - To ensure safety, use predefined validated content rather than artificial intelligence–generated responses [78].
  - Easy to access or understand [64,74,86], including by users with diverse computer skills [80].
  - Provide encouraging [72] and reassuring [76] words and display positive emotional expression [84].
  - Ask about and validate users’ feelings and make them feel heard [59-61,74,84,91].
  - Should be accessible and available 24/7 from anywhere [60,64,68,88].
  - Limit input options to reduce misunderstandings by MHCA [60,66,73,75,79,85].
  - Allow personalization of MHCA name, avatar, and personality to feel more safe [71,77].
  - Avoid repeated requests for the same private information [76].
  - Incorporate human-like elements such as autonomous variation in language that imitates human speech [84], approachable and personable tone [70,77], relational behavior [80], compassion [66], care [61], and the ability to identify implicit cues from the user [66].
  - MHCA should display empathy generally [12,61,63,64,77,85,86,92], through asking how the user feels [68] and providing responses customized to detected mood [74,92].
  - Do not send lengthy messages when users are in distress [60].
  - Provide options for silent interactions, such as text-based communication [81,84].
  - Provide content that is accurate, contextually appropriate, and based on expert or high-quality sources [60,64,68,70,86,91].
  - Ensure the MHCA is flexible and responsive to unexpected user input [12,61,70,92].
- Trustworthiness and transparency
  - Address users by name to support personalization and engagement [76,84].
  - Use active listening cues, such as brief affirmations or acknowledgments, to convey empathy and attentiveness [70] and do not program an avatar to look away, as it may convey untrustworthiness [79].
  - Consider using a visual depiction of the MHCA when appropriate but remain mindful of context and user comfort [82]; eg, same-race avatars may build trust but reduce willingness to self-disclose for some users [72].
  - Display high ability to tell right from wrong [66].
  - Clearly communicate data privacy and security practices, demonstrating how user data are protected and stored [67,68].
  - Be aware of the type of information stored and requested; users may trust MHCAs less to store more personal types of information [67].
  - Proactively address technical issues such as lagging, crashing, or difficult-to-follow navigation [59-61,64,67,72,73,83].
  - Provide coherent, relevant, accurate responses [59,60,64,78,91,93] in real time [87] and support translation between languages [91].
  - Incorporate a set structure in daily interaction sequences [81].
  - Provide personalized, nonrepetitive content [12,13,60,61,64,67,72,73,81,84,86]; comprehensive customization can help set realistic expectations about the MHCA [72].
  - Enable the MHCA to retain memory of prior conversations, enhancing continuity and personalization [59].
  - Offer relevant psychoeducational content to create transparency and address stereotypes against user conditions [85].
  - Provide example scenarios [70,76] and exercises [59] that are relevant and connected to users’ reported experiences.
  - Avoid overstating the MHCA’s technical capabilities [68].
  - Do not make the user feel inadequate or left out [66].
  - Ensure consistent and reliable presentation of outputs, maintaining uniformity in results and recommendations [61,76].
  - For audio-based MHCAs, include an option to repeat the last statement [75] and allow backtracking if something is mistakenly clicked [60].
  - Adopt a fluid, positive, and warm tone [66,70,76] while avoiding overly friendly behaviors such as “corny” language or "trying too hard” [66].
  - Be transparent about MHCA’s role, limitations, purpose, data handling [60,67,68,71,74,76,77,79,83,85,87], target audience [60], and free versus paid features [73].
  - Be transparent about MHCA’s nonhuman identity [72,74,77,84,87] by using a robotic or neutral name [76,77], cartoon avatar [79], and not pretending to have a human-like backstory [84].
  - Explain how to perform suggested activities, including why they were chosen, their purpose, and who created or assessed the information [59,60,64,70,76,77,91].
  - Provide technical information [75] and clearly instruct users on how to use and navigate the app from the beginning, eg, through a system orientation [59,60,73,83,90,91].
  - MHCA should be clear about what it knows about the user and permit modification of this information [71].
  - Indicate if the MHCA does not understand user input [60].
  - Incorporate regular, well-timed check-ins and reminders to support user accountability [12,62,70,74,92], encourage engagement in activities [85], and track mood [87] while allowing users to customize frequency and opt out [70].
- Collaboration and mutuality
  - Build trust and rapport so a user can express themselves freely [60], strengthening the therapeutic alliance with the user from the first interaction [69] by demonstrating care and interest in the user and establishing norms and expectations for interactions [79].
  - Use a pictorial character [77] or some elements of anthropomorphism [79] such as emojis and humor [85] (ie, to make it feel as though you’re talking to “someone”) as a shortcut for building rapport and working alliance with user.
  - Allow personalization of MHCA therapy style to increase agreement on therapy goals [71].
  - Allow users to set goals and select desired areas of focus [12,73,85].
  - Help users set goals and problem-solve by providing psychoeducation about setting goals, identifying goals with users, and setting reminders to check about goal completion [65].
  - Offer shared activities with the MHCA [84].
  - Offer initial survey to learn about users to provide more curated suggestions [76].
  - Provide a summary of the last interaction [59,85].
  - Call users by name to increase personal rapport [71,84]; this may decrease user privacy [76].
  - Avoid making MHCA overly prescriptive [81].
  - Foster individual autonomy to develop therapeutic alliance [78].
  - Include details in responses and ask follow-up questions to encourage users to vent [66].
  - Follow up through daily check-ins to promote accountability [12,66,74,92], remind them about previous tasks and activities [85], track mood [87], and make them feel cared for [70].
  - Compare old conversations regularly to track symptoms and update goals or treatment plans with users accordingly [86].
- Peer support
  - Find better ways to integrate group support [73].
  - Provide a “seek support group” feature [77].
- Empowerment, voice, and choice
  - Include diverse output options, including text, video, audio, and images [62,65,85,94], as well as diverse ways of visualizing user data [85].
  - Permit diverse input options [59,60,62,65,84,85], including free text [59,60,62,64,73,77,78,84,85,89,94], buttons or options [59,60,64,77,80,84,89], and speech [62,84,85].
  - Ability to opt out from MHCA reminders or notifications [63,92] and/or choose when these reminders are sent [62], avoiding checking in too often [70].
  - Be supportive, encouraging, and motivating [64,74,77,79] to instill users with a feeling that they are in control and things could change [60].
  - Ensure 24/7 accessibility to reduce feelings of helplessness [64].
  - Allow the user to lead the conversation [73,78,81,85] while sometimes taking the initiative to keep the conversation going [59].
  - Allow users MHCA customization [71,73,85], including aesthetic or cosmetic and functional customization [71,85] but consider what options users can customize and which are predefined, and have boundaries around customizable options [66].
  - Enable providing feedback [73] that personalizes interactions [81,91].
  - Input options should vary by task within the MHCA system [75].
  - Content should be available to view, download, and listen on demand [64].
  - For mood tracking, provide enough options to allow accurately capturing mood [83].
  - Provide specific and actionable help on condition-related tasks (eg, physician’s visits [77]).
  - Should allow users to connect with external apps and devices as they wish [13,62].
  - Enable selecting or creating MHCA avatar that makes user most comfortable [71,72,75].
  - Emphasize users achieved tasks [85].
  - Provide reports, graphs, or other visualizations on a regular basis to users recording their progress and mood [12,13,62,73,79,84,92] to facilitate reflection [12].
  - Give access to conversation history with the MHCA [77,80,90].
  - “See more” option for long messages [77].
  - Encourage users to deal with their problems [87] and take an active role in their care (eg, through cognitive behavioral therapy [85]).
  - Prioritize shorter and simpler activities [81].
  - Notifications and activity suggestions should be conscientious of users’ daily context and responsive to their reported emotions [81].
- Sensitivity to cultural, historical, and gender background
  - Support multiple languages to accommodate diverse users within a geographic region or country [60,83] and ensure intervention quality is retained when translating between languages [91].
  - If target users are not familiar with the terms the MHCA uses, provide a glossary [83].
  - Ensure racial mirroring is available for Black participants, as they had much stronger preferences for same-race agents than other racial groups [72].
  - Include a mixture of 2 or more mental health topics to be relevant to real-world users [86].
  - Provide text output alongside audio output to help nonnative language speakers or for noisy environments [80,81,84].
  - Visual output should be accessible in size and color for those with visual impairments [67].
  - Use audience-dependent language to indicate awareness of relevant audience-dependent topics [66,89].
  - Content should be diverse [64] and suit diverse populations [73].
  - Provide gender nonspecific, nonhuman MHCA to avoid triggering body image issues [77].
  - Avoid confrontation with users [66].

### Designing for Trust and Safety Through Availability, Anonymity, and Crisis-Ready Infrastructure

A core requirement across studies was building safe interaction spaces, where users felt unjudged and confident in how their data and disclosures were managed. Many studies emphasized that anonymity, whether through no registration models or optional pseudonyms, reduced fear of judgment and facilitated self-disclosure [60,68,78,83,88]. Conversely, using names was also found to build trust between the chatbot and the user [71,76], although it could raise privacy concerns [76]. Some studies bridged these concerns by recommending MHCA apps be password protected [89,90].

Similarly, transparent communication around data storage practices (eg, what is stored, for how long, and by whom) was tied to perceptions of trustworthiness [67,68,83,87]. Users were more willing to disclose information when they knew it was not archived indefinitely or shared across platforms [68,76]. These insights mapped onto the digital safety–related themes in Table 4. While secure data practices established the foundation of trustworthiness, usability, and availability further determined whether users experienced the MHCA as a dependable source of support. Several studies reiterated that chatbots should be accessible 24/7, enabling users to seek support from home or other safe spaces, reducing feelings of helplessness, and ensuring timely assistance [60,64,68]. Furthermore, interfaces should be intuitive and easy to navigate, addressing usability challenges [64,73] and providing usable, stigma-free emotional support [74].

Physical and emotional safety were further addressed when MHCAs incorporated crisis intervention mechanisms, such as save our souls (SOS) buttons, trigger-word detection, access to emergency helplines, and tailored resources for suicidal or self-harm–related disclosures [59,63,64,73,74,76,77,83,86,92]. Transparent statements clarifying that the CA is not a crisis service also supported trustworthiness by setting clear boundaries [74,87]. Together, these practices mirror themes related to safety, trustworthiness, and transparency outlined in Table 4.

### Crafting Human-Like, Empathic, but Transparent Behaviors

Users appreciated empathic, relational behaviors from MHCAs but still wanted clarity about their nonhuman identity. MHCA designs communicated their nonhuman nature, purpose, capabilities, and limitations upfront through robotic names or avatars, onboarding disclosures (eg, “digital coach vs therapist”), or welcome messages [68,74,77,79,83,85,87,89]. This transparency promoted user autonomy, confidence, and motivation, as users could engage with the system with clear expectations of its role and limitations [76]. Conversely, artificial backstories by chatbots, exaggerated friendliness, or attempts to appear human were often perceived as deceptive and diminished trust [66,84], highlighting the importance of neutral, “robotic” visuals, such as cartoon avatars [79]. While clarifying the MHCA’s nonhuman identity set the foundation for trustworthy engagement, users also relied on ongoing transparency within the interaction, including about data use and crisis handling. Providing clear instructions, menu navigation, and orientations helped users navigate the system efficiently, prevented misunderstandings, and supported self-efficacy and confidence in interacting with the MHCA [59,60,77,79,83,90,93]. One study also suggested that the user be able to view and modify what the MHCA had learned about the user to improve transparency [71]. Overall, transparency about data use and crisis management boundaries improved predictability, engagement, and trustworthiness, reflecting concepts of digital safety and responsible use [74,76,83].

Other relational design elements, such as checking in regularly [62,70], engaging in active listening [66,70], or expressing sympathy [74,76,85], supported positive emotional and psychological experiences of use. These features promoted emotional safety and enhanced user confidence, motivation, and comfort [68,74,85]. Well-designed avatars could further strengthen rapport and comfort, as user preferences modulated the effects of customized anthropomorphic versus neutral representations [72,79,82,84].

### Ensuring Accurate, Relevant, Nonrepetitive Responses to Build Trust and Reduce Distress

Designing for trustworthiness in MHCAs was closely tied to the system’s accuracy, reliability, and consistency. Some studies suggested that it is critical to provide clinically validated content where the materials are predefined and professionally reviewed, instead of generative AI outputs, to ensure all responses are validated, clinically safe, trustworthy, reliable, and of high quality [60,68,76,78,86]. MHCAs should clearly indicate whether individual pieces of content have been verified to enhance credibility [60,68,76]. Additionally, MHCAs should explain why specific interventions are selected and identify the source or author of self-care modules so users can properly understand and evaluate recommendations [76,77].

Receiving the right kind of help, accurate interventions, and contextually appropriate responses was critical for maintaining confidence in the system [60,64,68,70,76,78,91]. Technical issues, including glitches, slow loading, or failure to respond to unexpected user inputs, could undermine trust and engagement [59-61,64]. Similarly, inconsistencies in intervention delivery or errors in translating content (eg, English to Spanish) reduced perceived reliability [60,91]. Alongside reliability, adaptive and nonrepetitive content sustained user trust and perceived usefulness. Users valued responses that were tailored to their experiences, were not repetitive, and adapted dynamically based on feedback or prior interactions [13,60,61,64,70,76,81,84]. Incorporating user-specific language and ensuring real-time responses made interactions more understandable, relatable, and trustworthy [87,89]. Conversely, rigid or repetitive patterns or failure to remember prior conversations decreased trust and disrupted collaboration [59,64,86].

### Supporting User Autonomy via Control Over Settings, Interface, and Progress

Supporting user autonomy and empowerment through flexibility and personalization was another central theme. Several studies discussed the importance of users taking control of MHCA interactions and settings. For instance, allowing users to lead conversations [78] or choose areas of focus within an app, such as Wysa [73], increased autonomy and supported therapeutic alliance [78]. Scrolling back through past interactions [80,90], opting in or out of reminders [63,92], and determining the timing of check-ins [62] gave users control, promoting confidence and ownership over their healing.

Embedded customization and flexibility were also common themes across studies. Allowing users to personalize the personas of therapeutic agents [72], select intervention components [85], or modify avatars to reflect their preferences [71,75] created a sense of control, comfort, and trust. Similarly, giving users the option to translate speech to text [84], download or review chatbot content [64], and navigate flexibly through the system [60] promoted self-efficacy [66]. Systems that supported multiple modes of input and output (eg, text, free text, audio, video, clickable buttons, emojis, graphical interfaces, and PDF or figure uploads) further empowered users to engage in ways that matched their preferences and needs [59,60,64,84,85,88,89]. Alternatively, providing predefined options alongside free-text input, with clear protocols when input is not understood, balanced flexibility with safety [59,60,66]. Beyond MHCA settings and interface, designing shared goal setting, check-ins, and accountability mechanisms operationalized user empowerment and collaboration. For example, Woebot and similar agents allow users to set goals, track progress, and engage in regular check-ins [12,62,74,92]. Daily or weekly follow-ups and reminders help maintain engagement, provide structure, and signal that user input is valued, thereby indirectly reinforcing both collaboration and empowerment [84-86] alongside regular reports summarizing user mood, progress, or activity [12,13,62,73,79,85].

### Creating Context-Sensitive and Accessible Communication

Design recommendations related to sensitivity to cultural, historical, and gender issues included accessibility and responsiveness to user preferences, language, and characteristics. Users requested accurate multilingual support and content that reflects their lived realities, including gender, race, and age [60,72,73,77,83,91]. Implementing features such as a gender-nonspecific avatar [77], agents that mirrored the user’s race [72], youth-appropriate language, nontriggering naming options, and localization affected inclusivity, trustworthiness, and perceptions of safety [66,71,79]. Accessibility included specifics of basic, usable interface design, with users valuing readable fonts and clear icons [67], as well as nonverbal or text-only interaction options in noisy or vulnerable environments [80,84]. Context-aware behaviors, such as avoiding inappropriate suggestions when users are distressed, further supported emotionally responsive interactions, indirectly reflecting the idea of sensitivity [81].

## Discussion

### Positioning TIC in MHCA Research

Although SAMHSA’s TIC framework was originally developed for clinical contexts, it has been increasingly adapted in nonclinical domains such as educational institutions [96] and workplaces [97], and there is growing interest in applying TIC within HCI research [43,44,95]. Despite this uptake, the TIC framework has yet to be systematically applied to MHCAs, which is a relevant opportunity, given these systems’ interaction with users in vulnerable psychological states. This scoping review represents an initial effort to bridge clinical and technological domains, connecting research and practice across the heterogeneous MHCA literature. We chose to expand our analysis beyond mentions of explicit terminology to reflect the intrinsic subjectivity and interconnectedness of the TIC framework and to more comprehensively identify and summarize how scholarship to date has operationalized TIC-related ideas. In this section, we highlight key opportunities for future work to leverage the TIC framework in MHCA development, informed by the range of TIC-related language, constructs, metrics, and design recommendations identified in our review.

### Fragmented Engagement With TIC in Definition, Evaluation, and Design

Our scoping review of 38 publications reveals a nuanced and varied but fragmented landscape of MHCAs that implicitly reflects concepts aligned with the TIC framework. Many studies addressed safety (eg, anonymity, secure data practices, positive emotional experience, and crisis infrastructures); trustworthiness and transparency (eg, accuracy, reliability, signposting, and nonhuman identity disclosure); empowerment, voice, and choice (eg, control over settings, personalization, and flexibility); and collaboration and mutuality (eg, therapeutic alliance, check-ins, and accountability). Other TIC principles receive far more limited attention: peer support and sensitivity to cultural, historical, and gender issues were notably absent, not only in design but also in evaluation and theoretical foundations. Furthermore, while we identified design recommendations across studies that reflected TIC principles (eg, ensuring content quality, providing disclosure about MHCAs, offering control and customization), these recommendations were at times vague, emphasized different domains, or even presented contradictory guidance (eg, anonymity vs identity and customization, human-like vs clearly robotic). This reflects that developers lack standardized guidelines, checklists, or design matrices to systematically implement TIC principles. Finally, while it is encouraging that TIC-related constructs such as accuracy, consistency, security, therapeutic alliance, goal setting, and mutual respect are increasingly measured using validated quantitative scales (eg, WAI-SR, CUQ, and SUS), in many instances, TIC-related elements are relegated to background, discussion, and supplementary qualitative findings and were not central to studies’ or MHCAs’ purpose, approach, or design.

### Uneven Exploration of TIC Principles: Opportunities for Missed Principles to Fill Gaps

While large language models (LLMs) are gaining traction in mental health applications [98-100], most MHCAs in included publications were completely rule-based, inviting discussions about safety. The use of predefined, structured interactions over open-ended dialogue was often promoted in the corpus, highlighting current limitations in natural language processing capabilities, computational resources, and the need to minimize clinical risk from inappropriate or incoherent responses. Other prior work underscores that LLM-based CAs are not yet mature or reliable enough for psychologically and physically safe implementation in high-stakes, trauma-sensitive conversations [101,102]. From a trauma-informed perspective, recognizing acute vulnerability and preventing harm are foundational considerations.

Although some studies referenced crisis escalation or support mechanisms, this review did not systematically sample research explicitly addressing suicide risk. This limited our ability to evaluate how comprehensively MHCAs address safety and crisis vulnerability, particularly given that agents designed for depression and anxiety may interact with populations for whom suicidality is clinically relevant. Despite this, our review still surfaced a critical safety gap as half of the MHCAs in the corpus did not describe crisis intervention mechanisms or protocols. Overall, discussions about safety were scattered across paper sections, and a unified sense of real or perceived safety was rarely evaluated, raising concerns about how psychological and physical safety are truly ensured during MHCA interactions and echoing another recent systematic review that identified safety as underevidenced in many MHCA deployments [10]. Addressing these gaps may support the development of measurable, standardized requirements that promote both clinical effectiveness (eg, when using measures such as the Patient Health Questionnaire-9) and user safety, particularly for trauma-affected populations.

Crucially, too, our corpus included little engagement with cultural, historical, and gender issues, which influence many forms of traumatization and which are intended in the TIC framework to underlie and inform all parts of clinical and organizational practice [53]. Accessibility and usability, common focuses in included studies, can be implicitly connected to cultural, historical, and gender issues but are limited by the demographics of participants whose usability and access needs are represented. The influence of systemic and historical power dynamics on users’ experiences with MHCAs was largely unexplored, beyond relatively narrow references to demographic- or diagnosis-specific needs [62,79,88]. It is possible that researchers addressed these issues in practice in researcher-participant dynamics (eg, through institutional review board interactions) but did not report them in publications or did not incorporate them in MHCAs beyond study design ethics.

This represents a significant missed opportunity. Explicitly incorporating cultural, gender, and historical factors in MHCA design, research, and user experience could enhance intervention customization and user satisfaction [66,80], while also addressing pressing ethical challenges, such as user safety [27,103,104], LLM-related bias [27,105,106], access and literacy gaps [10,107,108], and the tools’ ability to handle nuance and complexity [103,104,109]. The issue of rule-based versus LLM-based interventions is also extremely salient for addressing sensitivity to cultural, gender, and historical factors. Depending on how they are trained or fine-tuned (which may frequently be entirely opaque), different LLMs can show differential orientations toward specific cultural values, displaying biases and reinforcing stereotypes [106] or struggling to appropriately handle race [105]. Algumaei et al [10] echoed this in their 2025 systematic review; they found that effective localization and cultural adaptability in MHCAs varied by implementation and that deficits in accessibility and inclusivity (especially for low-resource settings and Western-oriented systems) persist. Overall, the relative absence of potentially unsafe or biased LLM-based MHCAs, of written detail on MHCA interventions, and of historical sensitivity in the corpus is a critical gap where the TIC framework may provide guidance.

Finally, peer support was nearly absent from the corpus. For the 3 papers that mentioned it, recommendations were limited, with some suggestions that MHCAs encourage seeking out in-person support from friends and family [73,77,79] and that it might include a “seek support group” option [77]. Furthermore, it is theoretically unclear whether users perceive relationships with MHCAs as peer support and equally unclear how MHCA designers and researchers intend this relationship to be characterized. The fact that papers in our corpus variably used therapeutic alliance, friendship, or user tool to characterize the user-MHCA relationship, as well as the conflicting recommendations for human-like versus clearly robotic MHCA, warrants further attention. Scholarly work has criticized positioning MHCAs as *therapists* [27,28], but other relational dynamics, including MHCAs as *peers*, are less well-explored.

### Conceptual and Evaluative Discontinuities and Redundancies: Opportunities for TIC to Add Concise, Unifying Framework

A key finding of our analysis was that the field lacks a shared, digital CA-specific framework to justify and evaluate MHCA design and performance through a lens that is both trauma-informed and based in best clinical practice. The variety of terms and concepts (Table 3 and Table 4) and metrics (Multimedia Appendix 5) we identified as related to the TIC framework, in the absence of citing it directly or completely, results in inconsistent definitions, even when publications use the same terminology (ie, “trust” in the context of the Trust in Automation scale may be different than “trust” in the context of therapeutic alliance). This complicates researchers’ ability to identify patterns in quantitative outcomes and design recommendations, fragmenting interrelated ideas across CA-related domains and disciplines.

While we noted concepts *related* to TIC principles to be assessed completely or partially by an array of quantitative scales, this may also lead to redundancy, as multiple tools assess similar ideas but use differing theoretical framings or language. For example, the CUQ (used by Boyd et al [67]), designed to evaluate CAs, includes a question implicitly measuring its trustworthiness and safety: “Chatbot responses were useful, appropriate, and informative” [110]. The Client Satisfaction Questionnaire, as it was cited in a study by Liu et al [86], is validated for residential substance abuse treatment and includes a comparable question about usefulness: “Have the services you received helped you deal more effectively with your problem?” [111]. However, both questionnaires also include questions that do not overlap and were intended for different contexts, making overall CUQ or Client Satisfaction Questionnaire scores, or even answers to similar questions, incomparable. Without a holistic framework or metrics, it is difficult to build a clinically based, evaluable standard for MHCAs’ therapeutic experience that goes beyond clinical symptom outcome scores such as the Patient Health Questionnaire-9 or Generalized Anxiety Disorder-7 questionnaire.

However, it is not necessary to find one-to-one mappings of TIC principles. While fragmented, the interdisciplinarity of this topic area and the heterogeneous terminology we identified also illustrate many opportunities to adapt, combine, and validate constructs and measurements from a variety of fields, such as psychotherapy (eg, working alliance [112] and self-efficacy [113]), user experience design (eg, accessibility [114] and usability [115]), HCI (eg, Trust in Automation scale [76] and privacy and security [116]), and study of interpersonal relationships (eg, self-disclosure [117] and relational flourishing [118]). The body of research on explainable AI may provide use for increasing *trustworthiness* and *transparency* [119]. Drawing from critical studies, such as feminist [120], decolonial [121], and disability justice [122,123] approaches, may be useful for identifying relevant *cultural, historical, and gender issues* and beginning to address them. Scott et al [124] note that approaches to accountability and repair (eg, from restorative justice) echo TIC’s focus on those harmed; when MHCAs breach *safety, trust, transparency,* or fail to properly consider *cultural, historical, and gender issues,* these may also be highly relevant. Given the TIC framework’s American origin and interest in inclusivity, as well as well-known inequities in who MHCAs are developed with and for [10,107,108], future integrative development should include cultural, historical, and gender considerations that affect diverse MHCA users, not only those who are easy to reach or accommodate. In our corpus, age [68,70,73,77,84,91] and diagnoses and symptom patterns [62-64,75,79,88,92] were common sampling mechanisms. However, diagnosis and screening tools are often functions of culture and access [125-128], and salient demographic concerns in AI development also include gender [129], disability status [130], and more.

While the precise, practical, and preferred operationalization of the TIC framework and all its principles in the MHCA domain remains a question for future work, this scoping review has identified that an array of TIC-related ideas, regardless of their feasibility, are already present in the current academic landscape. The interconnectedness of the concepts we identified (eg, working alliance reflecting trustworthiness and transparency and collaboration and mutuality; customization reflecting empowerment, voice, and choice and collaboration and mutuality) suggests that going a step further to include more or all of TIC’s interrelated principles will likely be consolidating and descriptive; addressing one principle often directly or indirectly reinforces others. A more intentional synthesis, as well as filling the gaps noted earlier, under the umbrella of the TIC or trauma-informed computing framework, could help unify the inconsistencies we identified as well as inject empirical, clinical, and organizational best practices into the design of these implicitly relational digital health tools.

### Limitations

A key limitation of our scoping review is that no paper explicitly referenced the TIC framework or consistently defined its principles in the context of digital interventions. As a result, we had to infer alignment with TIC based on interpretation rather than author intent, introducing some subjectivity and internal biases in the results based on the authors’ specific backgrounds (ie, in HCI, public health, and user experience and not in applied TIC). Additionally, as only one study specifically focused on patients with trauma and many deliberately avoided including participants with severe mental health concerns, our ability to surface more nuanced design implications specific to trauma-affected users was constrained. This sampling tendency among publications also resulted in a marked absence of suicidality as a concern in MHCA and study design. We note that each aspect of TIC is highly complex; while our analysis intentionally adopted a holistic, scoping lens, each principle warrants future in-depth engagement. In particular, equity- and historical trauma–centered approaches may lead to different and more in-depth conclusions with regard to the differential impacts of MHCA design and deployment.

Furthermore, not all CAs used for mental health support are labeled as MHCAs, which may have excluded studies from this academic corpus. Many publications provided minimal detail on chatbot interfaces, workflows, or example interactions, limiting our ability to assess their alignment with TIC principles or determine whether systems were rule-based or AI-driven. Most MHCAs in our corpus were also prototypes, offering limited visibility into deployed or widely used systems. Finally, our review was limited to English-language publications and focused on the US-based SAMHSA TIC framework, which may have excluded relevant international or culturally specific approaches. Future research could broaden this scope by including gray literature, commercial documentation, and non-English sources, as well as considering alternative or complementary frameworks that reflect diverse global perspectives on trauma-informed design.

### Conclusions

The aim of this scoping review was to identify how the TIC framework is currently being adopted in the evaluation and design of MCHAs in academic literature. While no prior studies explicitly applied the TIC framework, we identified numerous instances where TIC-related concepts were referenced. Most commonly, these references were to trustworthiness and transparency; empowerment, voice, and choice; collaboration and mutuality; and safety, with peer support and cultural, historic, and gender issues largely overlooked. These principles were typically described in intervention design or discussion sections rather than rigorously evaluated. We also observed emerging design trends that support trauma-informed approaches, including customizability, consistency, flexibility, accuracy, and positive emotional expression. Collectively, these findings highlight opportunities to develop holistic, clinically informed, and trauma-informed design guidelines, metrics, and evaluation methods for MHCAs, providing a foundation for future interdisciplinary research in this space.

## Supplementary material

10.2196/77876 Multimedia Appendix 1 Detailed study metadata.

10.2196/77876 Multimedia Appendix 2 Mental health conversational agent metadata.

10.2196/77876 Multimedia Appendix 3 Detailed implicit elements.

10.2196/77876 Multimedia Appendix 4 Detailed design recommendations.

10.2196/77876 Multimedia Appendix 5 Quantitative scales used.

10.2196/77876 Checklist 1 PRISMA checklist.

## Abbreviations

ACM: Association for Computing Machinery
AI: artificial intelligence
CA: conversational agent
CBT: cognitive behavioral therapy
CUQ: Chatbot Usability Questionnaire
HCI: human-computer interaction
LLM: large language model
MHCA: mental health conversational agent
ML: machine learning
PRISMA-ScR: Preferred Reporting Items for Systematic Reviews and Meta-Analyses extension for Scoping Reviews
RE-AIM: Reach, Effectiveness, Adoption, Implementation, and Maintenance
SAMHSA: Substance Abuse and Mental Health Services Administration
SUS: System Usability Scale
TIC: trauma-informed care
WAI: Working Alliance Inventory
WAI-SR: Working Alliance Inventory, Short Revised
WAV-12: Working Alliance Inventory, Short form, Dutch version
